# A duplex real-time loop-mediated isothermal amplification assay for simultaneous detection of elephant endotheliotropic herpesvirus types 1 and 5

**DOI:** 10.3389/fvets.2025.1651182

**Published:** 2025-08-26

**Authors:** Chunzhong Xu, Ziyan Wang, Yongjuan Zhao

**Affiliations:** ^1^Shanghai Wild Animal Park, Shanghai, China; ^2^Shanghai Public Health Clinical Center, Fudan University, Shanghai, China; ^3^School of Medicine, Anhui University of Science and Technology, Huainan, China; ^4^Medical Laboratory of Taizhou Fourth People’s Hospital, Taizhou, China

**Keywords:** EEHV1, EEHV5, probe-based LAMP, Asian elephant, hemorrhagic disease

## Abstract

Elephant Endotheliotropic Herpesviruses (EEHVs) are the cause of a highly fatal haemorrhagic disease (HD) in elephants primarily affecting young Asian elephants (*Elephas maximus*) in both captivity and in the wild. Timely and accurate detection of EEHVs is crucial not only for antiviral and supportive treatment, but also for the prevention and control of the EEHV pandemic with proper preventive intervention measures, which requires a simple and rapid assay. EEHVs contain seven genotypes (EEHV1-7), and EEHV1, EEHV4 and EEHV5 are mainly circulating in Asian elephants. Here, we developed a rapid, sensitive and specific duplex real-time LAMP assay for simultaneous detection of EEHV1 and EEHV5. The assay exhibited remarkable specificity with no cross reactions with nine other herpesviruses, and high sensitivity with limit of detection (LOD) of 30 and 189 copies per 25 μL reaction for EEHV1 and EEHV5, respectively. The assay exhibits high reproducibility and can be completed within 30 min, significantly shorter than the qPCR assay. Clinical validation showed that the duplex assay had a 100% concordance with a previously described qPCR assay for 22 samples from Asian and African elephants. Furthermore, no EEHV1 and EEHV5 were detected in elephants at Shanghai Wild Animal Park. The novel duplex LAMP assay represents a particularly valuable POCT tool to facilitate the routine surveillance of EEHV1 and EEHV5 in captive and wild elephants.

## 1 Introduction

Elephant endotheliotropic herpesviruses (EEHVs) are significant pathogens threatening young Asian elephants (*Elephas maximus*) and African elephants (*Loxodonta africana*) (1). EEHV can cause severe fatal hemorrhagic disease (HD) with a mortality rate of up to 66–85% (2–4). For example, about 65% of young Asian elephant deaths in North American zoos or circuses during 1995–2015 were ascribed to EEHV-HD (5). Although viral shedding, transmission, and dissemination of EEHV remain unclear, clinically infected elephants exhibit symptoms such as lethargy, anorexia, lameness, colic, and diarrhea, with many succumbing to the disease within 1 to 7 days after symptom onset (6).

EEHVs belong to the family *Herpesviridae*, subfamily *Betaherpesvirinae*, genus *Proboscivirus*, and species *Elephantid betaherpesvirus* (1).^1^ To date, seven EEHV genotypes (EEHV1, EEHV2, EEHV3, EEHV4, EEHV5, EEHV6 and EEHV7) have been identified, and five genotypes (EEHV1, EEHV3, EEHV4, EEHV5 and EEHV7) were further classified into two subtypes A and B (e.g., EEHV1A and 1B) (2). Because of lack of *in vitro* EEHV culture systems, currently there is no traditional vaccines, such as inactivated vaccines and live attenuated vaccines, as well as antiviral drugs against EEHVs (7). New vaccines, such as mRNA vaccines, are still in the experimental stage, and have not yet been fully developed for commercial use (7). EEHV-HD has a rapid disease progression resulting in irreversible cellular damage (3). Unsuccessful treatment of EEHV-HD cases might be often associated with delay in treatment (3). Rapid and timely diagnosis of EEHVs and routine EEHV screening tests are critical not only for antiviral (with drugs against other herpesviruses such as HSV-1 and HSV-2) and supportive treatment (3), but also for the prevention and control of the EEHV outbreak with proper preventive intervention measures (4).

Real-time quantitative PCR (qPCR) method is often used as gold standard for diagnosis of infectious diseases due to its high sensitivity and specificity (8). Several qPCR assays were previously developed for detection of EEHV1 (9–11). However, the requirement of qPCR assays for specialized equipment, laboratory facilities, and trained personnel limit their capacity for rapid point-of-care testing (POCT) in the wild and/or in the zoo. Therefore, rapid, sensitive, and cost-effective POCT assays will facilitate the surveillance of EEHVs in wild and captive elephants.

**Figure 1:**
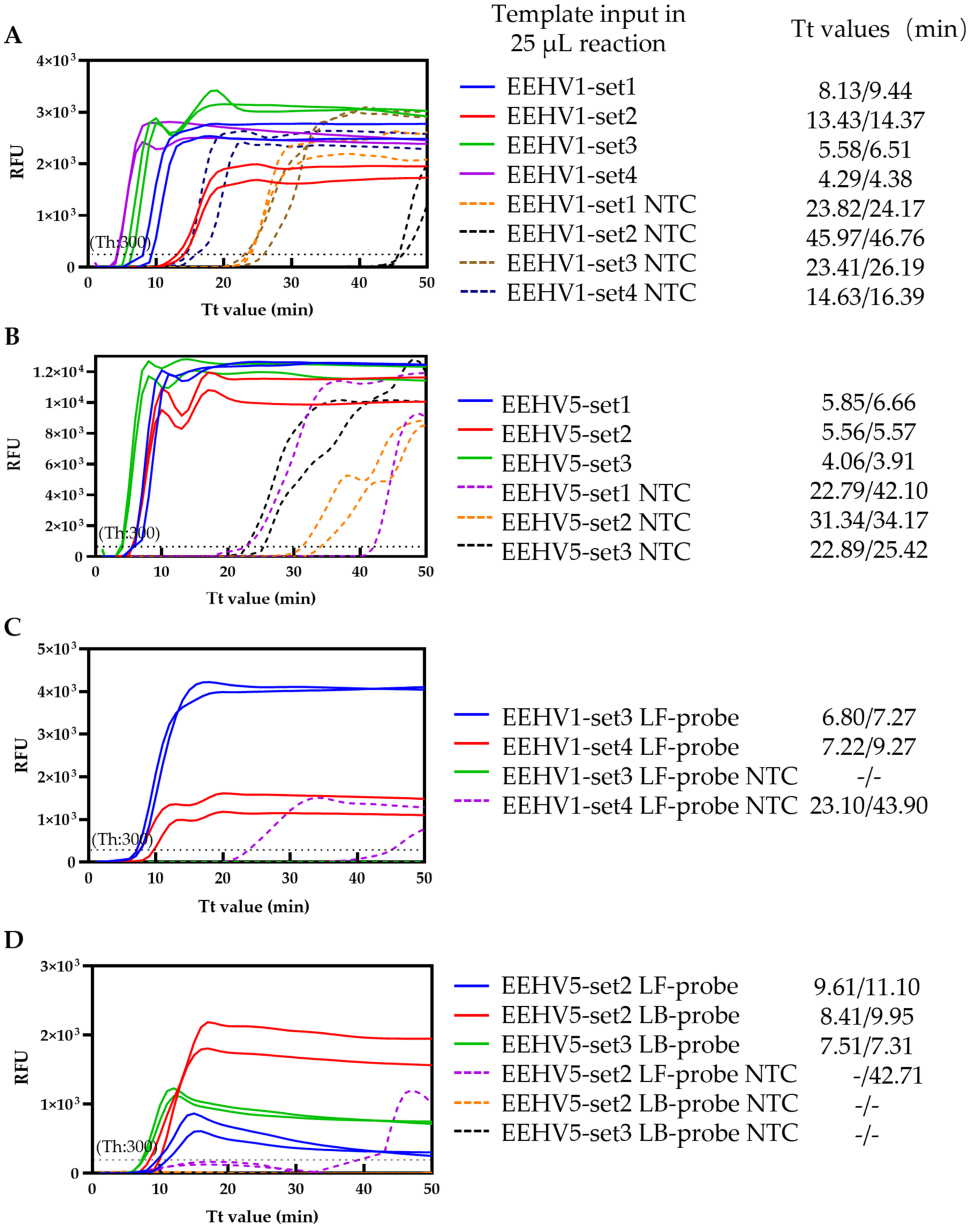
Screening of the optimal primer sets and probes. **(A,B)** Screening of the primer sets for EEHV1 and EEHV5, respectively. **(C,D)** Screening of the probes for EEHV1 and EEHV5, respectively. NTC, Non-template control; Th, Fluorescence threshold.

Loop-mediated isothermal amplification (LAMP) is a promising isothermal amplification technique for POCT diagnosis and offers simplicity, sensitivity and speed (12, 13). LAMP is often performed with pH-sensitive indicators (e.g., Cresol red), hydroxy naphthol blue (HNB), or fluorescent detection reagent (e.g., calcein) for naked eye visualization, or sequence-independent fluorescent dyes (e.g., SYBR green, SYTO 9) for real-time monitoring (14, 15). A LAMP assay with calcein was previously developed to detect EEHV1 (16, 17). However, frequent non-specific amplification by LAMP results in false positive results when above-mentioned indicators or sequence-independent fluorescent dyes are used (Figures 1A,B) (15). To overcome the limitation of non-specific amplification in molecular diagnosis, several probe-based LAMP methods have been developed and enable single tube multiplex detection of various targets (14, 18, 19). In this study, we developed a probe-based duplex real-time LAMP assay for simultaneous detection of both EEHV1 and EEHV5 and evaluated the novel assay using serum samples from elephants. The assay exhibits high specificity and sensitivity of the assay with limits of detection (LOD) of 30 and 189 per 25 μL reaction for EEHV1 and EEHV5, respectively, and showed an 100% concordance with a previously described qPCR assay in clinical evaluation.

## 2 Materials and methods

### 2.1 Materials

pUC57 plasmids containing the U38 gene of EEHV1 (GenBank: MW015025.1, nt: 92552–93,037) and EEHV5 (GenBank: NC_024696.1, nt: 86742–86,961) were synthesized by General Biotech (Anhui, China). The primers and probes were synthesized by Sangon Biotech (Shanghai, China). Bst 3.0 DNA polymerase, 10 × isothermal amplification buffer, and 100 mM MgSO_4_ were purchased from HaiGene Biotech Co., Ltd. (Haerbin, China). Deoxynucleotide solution mix (dNTPs: 10 mM each) were purchased from Genewiz (Suzhou, China). TIANamp virus DNA/RNA extraction kit was obtained from Tiangen Biotech (Beijing, China). Premix Ex Taq™ (Probe qPCR) Kit was obtained from Takara Bio (Tokyo, Japan).

### 2.2 LAMP primers and probe design

All available EEHV1 (66 sequences) and EEHV5 (16 sequences) genomic sequences were downloaded from the GenBank database and aligned with MEGA 10. Based on U38 sequences, three sets of LAMP primers were designed for EEHV1 and EEHV5 each using the PrimerExplorer V5 software (Supplementary Table 1).^2^ Another set of primers for EEHV1 (EEHV1-set4) was retrieved from a previous paper.

We first tested all four primer sets for EEHV1 and three sets for EEHV5 to find the primer sets with the highest amplification efficiency as previously described (20). Because of frequent non-specific amplification (false positive) in LAMP reactions with sequence-independent intercalating dyes or pH-sensitive indicators, probes were designed and used in the LAMP to improve the specificity (Supplementary Table 1).

In general, the sequence of the probe is identical to one of the forward or backward loop primers (LF or LB). If the use of probe does not completely avoid nonspecific amplification signal in the non-template control (NTC), the probe was further adjusted according to its binding region, Tm value, and secondary structure. Self-complementarity (more than 3 base pair interactions) and strong 3′ end complementarity are not allowed in the probe. DNAMAN software was used to predict the second structure of the probe.

### 2.3 Preparation of DNA standard

The pUC57 plasmids containing the U38 gene was used as DNA standard and quantified with a NanoDrop 2000C spectrophotometer (Thermo Fisher Scientific, United States). The copy number of the plasmid standard was calculated using the formula:


$$\text{dsDNA copies}/\mathrm{mL}=\frac{\mathrm{DNA}\;\text{concentration}\;(g/\mathrm{mL})}{\mathrm{DNA}\;\text{length}\times 660}\times 6.022\times 10^{23}$$

### 2.4 Dye-based LAMP and probe-based real-time LAMP reactions

The dye-based LAMP reaction was used to screen the optimal primer set. A standard 25 μL reaction contained 8 U Bst 3.0 DNA polymerase, 1 × isothermal amplification buffer, 6 mM MgSO_4_, 1.4 mM dNTPs each, 0.1 μM each of F3 and B3, 1.0 μM each of FIP and BIP, 0.6 μM each of LF and LB and 0.4 mM SYTO-9.

In the probe-based LAMP reaction, 0.3 μM probe was used to replace SYTO-9 in the conventional real-time LAMP reaction and the concentration of the loop primer that shares identical sequence to or overlaps sequence with the probe was halved from 0.6 μM to 0.3 μM. In the duplex real time LAMP assay for simultaneous detection of EEHV1 and EEHV5, the concentrations of all primers and probes for each virus were halved.

All real-time LAMP reaction were performed at 64°C for 50 min with real-time monitoring each minute using the CFX 96 Touch real-time PCR Detection System (Bio-Rad Laboratories, Hercules, CA, United States). Fluorescent signal was collected in SYBR Green channel every minute for the real-time LAMP reactions with SYTO-9, or collected in corresponding fluorescent channels every minute for the probe-based LAMP reaction. Threshold time (Tt) was automatically generated by the real-time PCR machine.

### 2.5 Sensitivity and specificity

To determine the specificity of the probe-based LAMP assay for detection of EEHV1 and EEHV5, nine herpesvirus strains, including human herpesvirus 3 (HHV-3), human herpesvirus 4 (HHV-4), human herpesvirus 5 (HHV-5), human herpesvirus 6B (HHV-6B), pseudorabies virus (PRV), bovine herpesvirus 1 (BoHV-1), bovine herpesvirus 4 (BoHV-4), canine herpesvirus 1 (CHV-1), feline herpesvirus 1 (FHV-1), were purchased from Bena Culture Collection (Henan, China). The concentrations of these virus strains were 10^5^–10^6^ copies/mL.

To determine the detection capacity of the probe-based LAMP assay, ten-fold dilutions of DNA standards, ranging from 3 × 10^4^ to 3 copies per 25 μL reaction.

### 2.6 Limit of detection and intra-assay and inter-assay variabilities

Sixteen replicates of serial dilutions of the DNA standards (3,000, 600, 120, 24 to 5 copies per 25 μL reaction) were used to determine the LOD of the duplex real-time LAMP assay for detection of EEHV1 and EEHV5 and to assess the intra-assay variability. The LOD was defined as the lowest detection concentration at 95% posterior probability using the probit regression analysis in SPSS 17.0 software. The intra-assay variability was analyzed by F-test. To assess the inter-assay variability, three independent experiments with 8 replicates (3,000 copies/reaction) were conducted on different days and the coefficient of variation (CV) was calculated using the following equations.


$$\mathrm{CV}(\%)=\frac{\text{Standard deviation}}{\text{Overall mean}}\times 100\%$$

### 2.7 Clinical evaluation

To assess the performance of the duplex real-time LAMP assay, three EEHV1-positive nucleic acid samples from Asian elephants were obtained from Guangzhou Animal Park. Nineteen serum samples previously collected from 16 Asian elephants and 3 African elephants raised in Shanghai Wild Animal Park for routine physical examinations were also used in the evaluation. The use of serum samples from elephants was approved by the Laboratory Animal Welfare & Ethics Committee of Shanghai Public Health Center (2025-A039-01). The study was compliant with all relevant ethical regulations regarding animal research.

Viral DNA was extracted from 200 μL serum samples using a commercial DNA extraction kit (TIANamp virus DNA/RNA extraction kit, Tiangen Biotech, Beijing, China) and eluted in 50 μL of nuclease-free water. The nucleic acid concentrations of these samples were 10–50 ng/μL by quantifying with a NanoDrop 2000C spectrophotometer (Thermo Fisher Scientific, United States). Five μL DNA was used in the duplex real-time LAMP assay. Two previously reported qPCR assays for EEHV1 and EEHV5 were carried out as gold standard (11, 21) and their primers and probes are shown in the Supplementary Table 2. The qPCR assay was performed using the Premix Ex Taq™ (Probe qPCR) Kit (Takara Bio, Tokyo, Japan). The concordance rate was calculated using the formula: (number of consistent results by both methods/total number) × 100%.

## 3 Results

### 3.1 Establishment of the duplex real-time LAMP assay

To establish the duplex real-time LAMP assay for the detection of EEHVs, we firstly screened the optimal primer set using the conventional LAMP method with SYTO™ 9 from four (including one set from a previous study) and three sets of LAMP primers for EEHV1 and EEHV5, respectively (Supplementary Table 1). The selection was based on the fastest amplification efficiency for specific target and minimal non-specific amplification signal in NTC (20, 22). The EEHV1-set 3 and EEHV1-set 4 showed higher amplification efficiency (Tt values: 5.58 and 4.29 min, respectively) than the other two primer sets (Tt values: 8.13 and 13.43 min) (Figure 1A). Therefore, the EEHV1-set 3 and EEHV1-set 4 were selected for probe design albeit the latter had higher non-specific amplification signal (Tt values: 14.63 min) than the former (Tt values: 23.41 min) in NTC. Similarly, the EEHV5-set 2 and EEHV5-set 3 were also selected for probe design due to their relatively faster amplification (Tt values: 5.56 and 4.06 min respectively) for specific target but less non-specific amplification signal in NTC (Tt values: 31.34, and 22.89 min) (Figure 1B).

**Figure 2:**
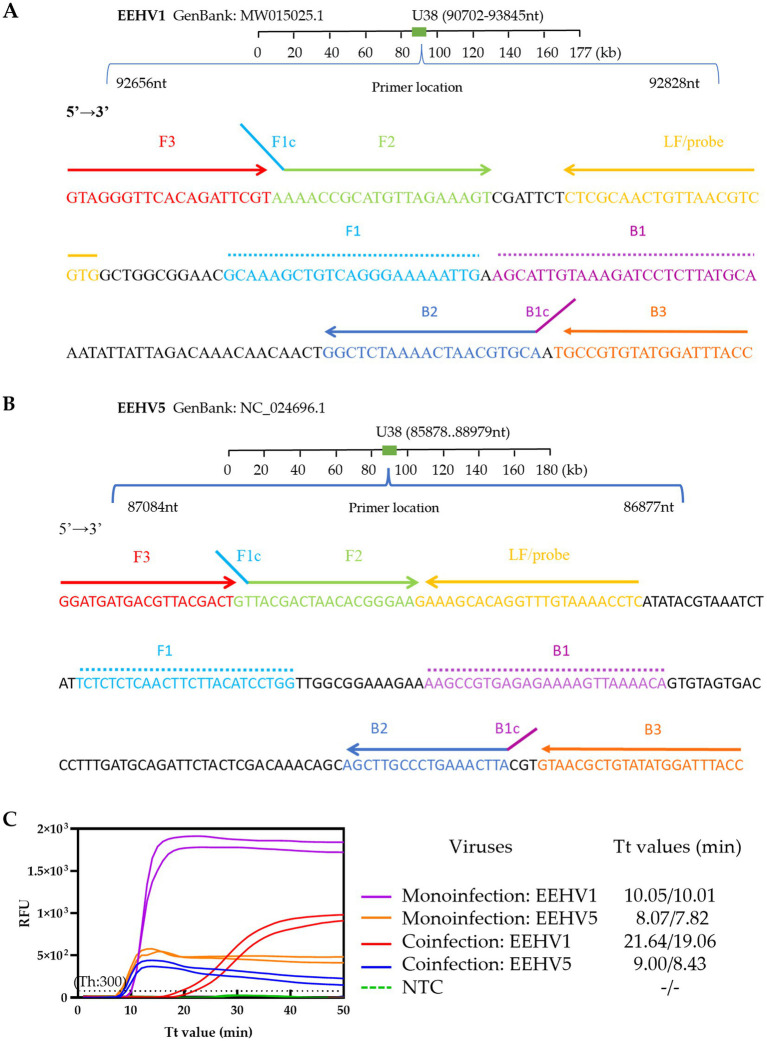
Establishment of the duplex real-time LAMP assay for simultaneous detection of EEHV1 and EEHV5. **(A,B)** Designs of the singleplex real-time LAMP assay for EEHV1 and EEHV5, respectively. **(C)** Performance of the duplex real-time LAMP assay in the detection of Mono-infection (individual plasmid standard) and coinfection (mixed plasmid standards) of EEHV1 and EEHV5. NTC, Non-template control; Th, Fluorescence threshold.

LF-probes were designed for EEHV1-set 3 and EEHV1-set 4 (Supplementary Table 1) and subjected to the real-time LAMP reaction. Slightly lower Tt values were obtained in the reaction with EEHV1-set 3 LF-probe (6.8 and 7.27 min) than that with EEHV1-set 4 LF-probe (7.22 and 9.27 min). Importantly, non-specific amplification signal was observed in the reaction with EEHV1-set 4 LF-probe (Tt values: 23.1 and 43.9 min) (Figure 1C). Therefore, EEHV1-set 3 containing LF-probe was selected in the real-time LAMP assay for the detection of EEHV1 (Figure 2A).

For EEHV5, LF-probe and LB-probe were designed for EEHV5-set 2 and LB-probe for EEHV5-set 3 (Supplementary Table 1). Comparative experiment showed that the EEHV5-set 3 LB-probe had better performance with lower Tt values (7.51 min) for specific targets and no non-specific amplification signal, whereas the two probes of the EEHV5-set 2 showed slightly lower amplification efficiency or yielded non-specific amplification signal (Figure 1D). Therefore, EEHV5-set 3 containing LB-probe was selected in the LAMP assay for the detection of EEHV5 (Figure 2B).

We performed alignments of all available EEHV1 and EEHV5 U38 gene sequences to assess the sequence conservation of the EEHV1-set 3 and EEHV5-set 3. All primers and probes of the two primer sets are highly conserved and cover almost the vast majority of the EEHV1 and EEHV5 variants (Supplementary Figure 1). Based on the results of the single LAMP assays (Supplementary Figure 2), a novel duplex real-time LAMP assay was established for simultaneous detection of both EEHV1 and EEHV5. We validated the assay using plasmid standards. The duplex assay showed good capacity for detecting mono-infection (individual plasmid standard) and coinfection (mixed plasmid standards) of EEHV1 and EEHV5 (Figure 2C). However, we observed that the duplex assay had a slightly lower amplification efficiency for EEHV1 (Tt: 19.06 and 21.64 min) than EEHV5 (Tt: 8.43 and 9.00 min) in the presence of same amount of both viruses in spite of higher fluorescence signal for EEHV1 than EEHV5 (Figure 2C).

### 3.2 Specificity and sensitivity of the duplex real-time LAMP assays

**Figure 3:**
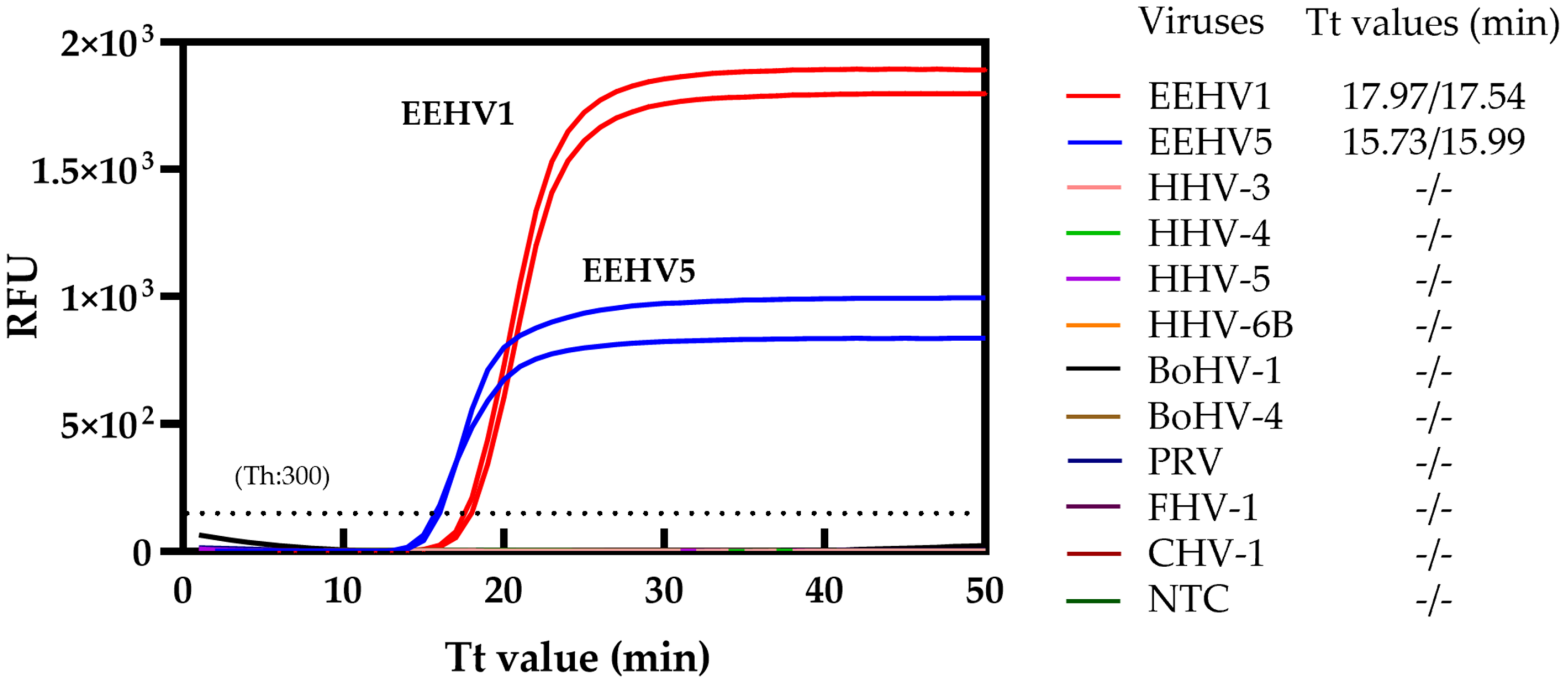
Specificity of the duplex real-time LAMP assay. NTC, Non-template control; Th, Fluorescence threshold.

To assess the specificity of the duplex real-time LAMP assay for both EEHV1 and EEHV5, nine herpesviruses, including HHV-3, HHV-4, HHV-5, HHV-6B, PRV, BoHV-1, BoHV-4, CHV-1, and FHV-1, were tested. No cross-reactions were observed for all these nine herpesviruses (Figure 3), supporting the high specificity of the duplex real-time LAMP assays.

**Figure 4:**
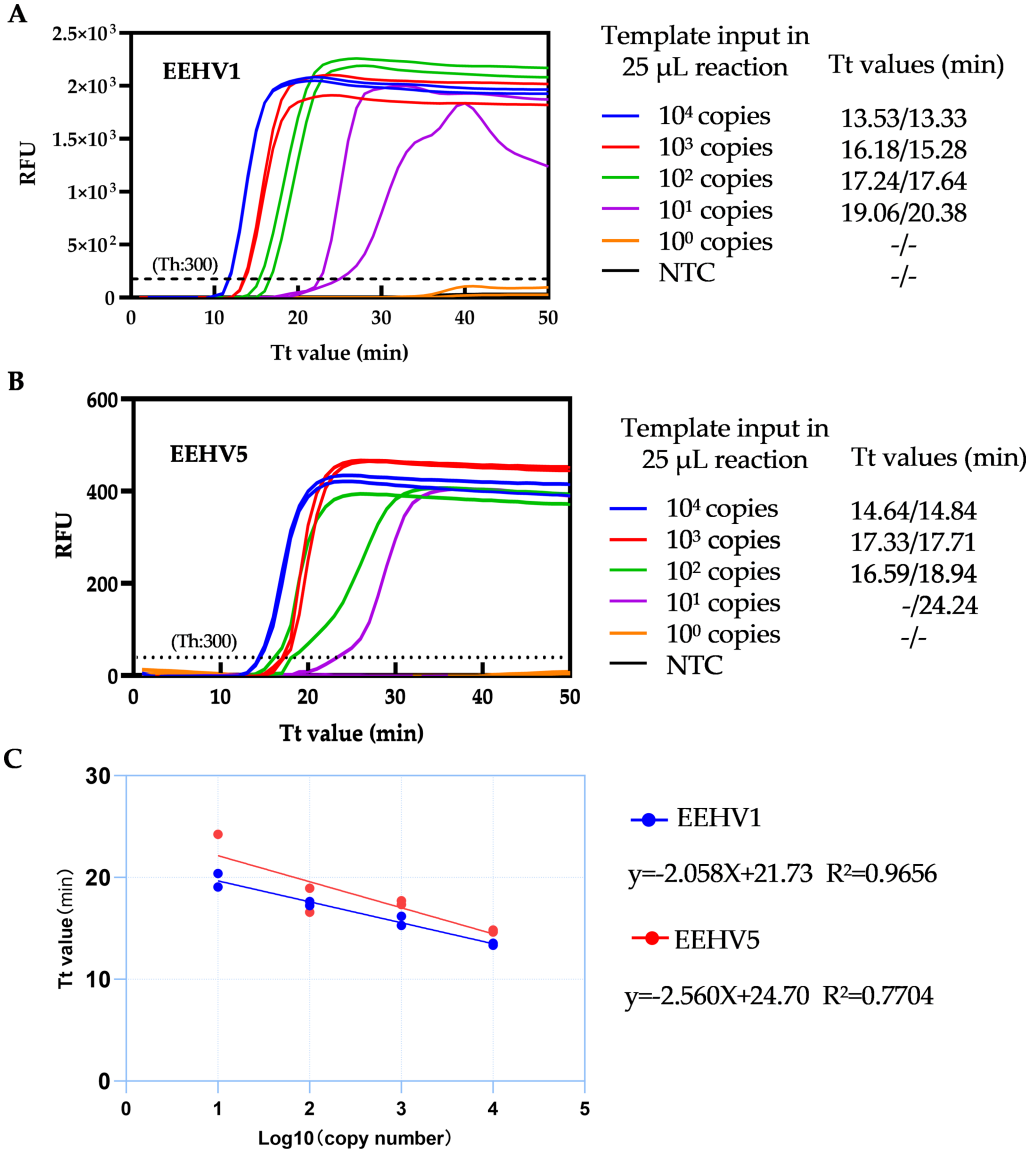
Detection capacity of the duplex real-time LAMP assay for EEHV1 **(A)** and EEHV5 **(B)**. **(C)** Linear correlation between Tt values of duplex real-time LAMP assay and the log_10_ copy number of template input. NTC, Non-template control; Th, Fluorescence threshold.

To determine the sensitivity of the duplex LAMP assay, we first performed the tests using EEHV1 and EEHV5 DNA standards from 10^4^ to 1 copies by ten-fold serial dilutions. The single real-time LAMP assay can detect 30 copies of EEHV1 and 3 copies of EEHV5 per 25 μL reaction within 30 min (Supplementary Figure 3). The duplex real-time LAMP assay can detect 30 copies of both EEHV1 and EEHV5 per 25 μL reaction within 30 min (Figures 4A,B and Supplementary Figure 4). Furthermore, a linear relationship between Tt values and the log10 copy numbers was observed (EEHV1: *R*^2^ = 0.9656, EEHV5: *R*^2^ = 0.7704) (Figure 4C).

We further determined the sensitivity of the duplex assay using 16 replicates of 5-fold serially diluted DNA standards of EEHV1 and EEHV5 from 3,000 to 5 copies per 25 μL reaction. For EEHV1, all 16 replicates were detected positive at the template input of 120 copies, and 14 replicates positive at the template input of 24 copies (Table 1). For EEHV5, all 16 replicates were detected positive at the template input of 600 copies, and 10 replicates positive at the template input of 120 copies. The LOD of the duplex real-time LAMP assay was estimated to be 30 and 189 copies per 25 μL reaction for EEHV1 and EEHV5, respectively (Table 1).

**Table 1:** Limit of detection (LOD) of the duplex real-time LAMP for EEHV1 and EEHV5 detection.

Template input (copies/25 μL reaction)	3,000	600	120	24	5	LOD (copies/25 μL reaction)
EEHV1 (positive/total)	16/16	16/16	16/16	14/16	6/16	30
EEHV5 (positive/total)	16/16	16/16	10/16	2/16	0/16	189

### 3.3 Repeatability testing

To assess the repeatability of the novel duplex LAMP assay for the detection of both EEHV1 and EEHV5, we performed intra-assay and inter-assay using eight replicates at three different days. The intra-assay and inter-assay CVs were 3.0 and 4.1% for EEHV1 and 3.5 and 5.1% for EEHV5, respectively (Table 2). These results indicate that the novel duplex LAMP assay has excellent reproducibility.

**Table 2:** Intra-assay and inter-assay reproducibility test of the duplex real-time LAMP assay.

Concentration of standard plasmids (copies/reaction)	Intra-coefficient of variation			Inter-coefficient of variation		
	*n*	mean±SD	CV%	*n*	mean±SD	CV%
EEHV1 (3,000copies/reaction)	8	12.53 ± 0.37	3.0	3	12.14 ± 0.50	4.1
EEHV5 (3,000copies/reaction)	8	9.31 ± 0.33	3.5	3	8.87 ± 0.45	5.1

### 3.4 Evaluation of the multiplex real-time LAMP using clinical samples

The application of the duplex real-time LAMP assay was assessed using 22 clinical samples including 3 previously identified EEHV1 positive nucleic acid samples and 19 sera collected from elephants at Shanghai Wild Animal Park. A previously described qPCR assay was performed in parallel as gold standard (13, 14). All three EEHV1 positive nucleic acid samples were detected positive for EEHV1 by both the duplex LAMP and the qPCR assays (Table 3). All the other 19 sera were detected negative for EEHV1 and EEHV5 by the two assays, indicating that at least no EEHV1 and EEHV5 are prevalent in Shanghai Wild Animal Park. The concordance rates of the duplex LAMP were 100% with the qPCR assays (Supplementary Table 3).

**Table 3:** Comparison of the performance of the duplex real-time LAMP assays and the qPCR assays for 22 clinical samples.

Animal	Sample ID	EEHV1		EEHV5	
		duplex LAMP (Tt value)	qPCR (Ct value)	duplex LAMP (Tt value)	qPCR (Ct value)
Asian elephant	1–16	Neg.	Neg.	Neg.	Neg.
	20	14.72	29.70	Neg.	Neg.
	21	12.57	29.19	Neg.	Neg.
	22	10.31	22.68	Neg.	Neg.
African elephant	17–19	Neg.	Neg.	Neg.	Neg.

## 4 Discussion

International Union for Conservation of Nature (IUCN) Red List has categorized Asian elephants as endangered globally (23). Haemorrhagic disease caused by EEHVs is a leading cause of mortality among young Asian (*Elephas maximus*) and African (*Loxodonta africana*) elephants worldwide (4). Early detection through routine screening is critical for timely intervention (2). The Department of Wildlife and National Parks (PERHILITAN) of Malaysia, a government wildlife management authority, has established the National Elephant Conservation Action Plan (NECAP 2.0), including the implementation of an EEHV monitoring program (24). EEHV-HD is characterized by rapid disease progression (25). Early and timely antiviral and supportive treatments can decrease the risk of death from EEHV-HD, making early and accurate diagnosis of EEHV infection essential. Furthermore, frequent surveillance of EEHVs has been recommended for young elephants under 8 years of age. Routine testing of blood DNA from at-risk Asian elephants for EEHV viremia has been suggested to help prevent fatal HD and/or initiate treatment earlier (26). Therefore, there is an urgent need for simple, rapid, sensitive and accurate diagnostic assay for the detection of EEHVs.

Despite their high sensitivity and specificity, various qPCR-based assay may not be the optimal tools for rapid and cost-effective diagnosis of EEHVs in elephant populations in zoos, camps, and conservation parks due to its high dependence on expensive equipment, Lab condition and skilled personals, as well as time-consuming nature (27). Two LAMP assays were previously developed for the visual detection of EEHV1 with hydroxy naphthol blue (HNB), a metal ion indicator (16, 17). Non-specific amplification is common in LAMP reaction requiring three pairs of primers and is difficult to be overcomed via the optimization of primer design (28). The use of sequence-independent metal ion indicator can result in false positive results, which may limit the application of previous LAMP assays.

The use of probes in LAMP can largely improve its specificity and enable single-tube multiplex detection (29). In this study, we developed a duplex real-time LAMP assay for simultaneous detection of EEHV1 and EEHV5 using a previously described strategy. The assay has LODs of 30 and 189 copies of viral DNA per 25 μL reaction for EEHV1 and EEHV5, respectively, with no cross reactivity with other nine herpesviruses. Furthermore, the assay exhibits high reproducibility and 100% concordant with the established qPCR assays.

EEHV1 was the most predominant genotype responsible for EEHV-HD in young Asian elephants aged 1–4 years, accounting for about 90% of cases (30). The first fatality associated with EEHV5 was described in 2011 (31). EEHV5 was recommended for inclusion in virological diagnostics of Asian elephants (21). EEHV5 was included with EEHV1 in the novel duplex LAMP assay. However, lower detection sensitivity for EEHV5 was observed in the duplex system than that in single LAMP reaction despite further optimization of the concentrations of primers and probes. The main reason is that the multiplex system may slightly decrease the detection sensitivity of certain targets albeit the probe-based LAMP method has the potential for triplex detection (18, 29). Some studies showed EEHV4 has a slightly higher prevalence than EEHV5 in Asian elephants (3, 4). Non-inclusion of EEHV4 in the multiplex LAMP assay is one limitation of this study.

Apart from blood samples, previous studies showed that EEHV1, EEHV4 and EEHV 5 can also be detected in trunk and oral swabs of Asian elephants (26, 32). Swab samples are suitable for extraction-free detection by various POCT methods. The probe-based LAMP method was demonstrated to be well tolerant to swab samples and even blood samples (18, 22, 29). In view of the feasibility of trunk and oral swabs, and the nature of fast amplification, the extraction-free format of the novel duplex LAMP assay can facilitate the routine monitoring of EEHVs. However, lack of EEHV-positive trunk and/or oral swabs limited our ability to develop and assess the extraction-free duplex LAMP assay for the detection of EEHV1 and EEHV 5, which is another limitation of this study.

## 5 Conclusion

We developed a rapid, sensitive and specific duplex LAMP assay for simultaneous detection of both EEHV1 and EEHV5. The assay has LODs of 30 and 189 copies per 25 μL reaction for EEHV1 and EEHV5, respectively, and can be completed within 30 min. Clinical evaluation showed a 100% concordance between the novel duplex LAMP assay and the qPCR assay, and no prevalence of EEHV1 and EEHV5 was detected at Shanghai Wild Animal Park. The novel duplex LAMP assay represents a particularly valuable POCT tool to facilitate the routine surveillance of EEHV1 and EEHV5 in captive and wild elephants.

## Data Availability

The original contributions presented in the study are included in the article/Supplementary material, further inquiries can be directed to the corresponding author/s.

## Footnotes

^1^ https://talk.ictvonline.org/taxonomy/

^2^ https://primerexplorer.eiken.co.jp/e/
