# Supplementary material for: A duplex real-time loop-mediated isothermal amplification assay for simultaneous detection of elephant endotheliotropic herpesvirus types 1 and 5
Source: Front Vet Sci. 2025 Aug 26;12:1651182. doi: 10.3389/fvets.2025.1651182 (PMC12417173; doi:10.3389/fvets.2025.1651182)
Supplement: Supplementary file 1 [file Supplementary_file_1.docx]

Supplementary Material

**Supplemental Table 1.** Primers and probes used for LAMP assay in this study

| Viruses | Primer sets | Primers/probes | Primer sequence (5'-3') | Length (nt) | Refs. |
| --- | --- | --- | --- | --- | --- |
| EEHV1 | EEHV1-set 1 | F3 | AGTATCATTCAAGCATACAACTT | 23 | This study |
|  |  | B3 | GAGGATCTTTACAATGCTTCAA | 22 |  |
|  |  | FIP | GGTGGTGACCATTGTAACATCCCTTTGATCACAGATAATGATGTAGC | 47 |  |
|  |  | BIP | GATTCGTAAAACCGCATGTTAGAAATTTCCCTGACAGCTTTGC | 43 |  |
|  |  | LB | TTCTCTCGCAACTGTTAACGTCGT | 24 |  |
|  | EEHV1-set 2 | F3 | GAAGCATTGTAAAGATCCTCTT | 22 | This study |
|  |  | B3 | CAATCGTTAAATCGGTCACAA | 21 |  |
|  |  | FIP | CGGTAAATCCATACACGGCATTGTATTATTAGACAAACAACAACTGGC | 48 |  |
|  |  | BIP | TGTGTCGAAAGGGATGTTTCCCTTTGGTAACAGCTAGCAGT | 41 |  |
|  |  | LB | GGCCATAGCTGAGTCGGT | 18 |  |
|  | EEHV1-set 3 | F3 | GTAGGGTTCACAGATTCGT | 19 | This study |
|  |  | B3 | GGTAAATCCATACACGGCA | 19 |  |
|  |  | FIP | CAATTTTTCCCTGACAGCTTTGCAAAACCGCATGTTAGAAAGT | 43 |  |
|  |  | BIP | AGCATTGTAAAGATCCTCTTATGCATGCACGTTAGTTTTAGAGCC | 45 |  |
|  |  | LF | CACGACGTTAACAGTTGCGAG | 21 |  |
|  |  | LF-probe | BHQ2-CACGACGTTAACAGTTGCGAG-Cy5 | 21 |  |
|  | EEHV1-set 4 | F3 | TTGATCACAGATAATGATGTAGCCT | 25 | [1]. |
|  |  | B3 | GCACGTTAGTTTTAGAGCCAG | 21 |  |
|  |  | FIP | CGACTTTCTAACATGCGGTTTTACGAGAGGAGGATGTTACAATGGTCA | 48 |  |
|  |  | BIP | CTCTCGCAACTGTTAACGTCGTGCATAAGAGGATCTTTACAATGCTTC | 48 |  |
|  |  | LF | ACCCTACCGGTGTTGGTGG | 19 |  |
|  |  | LB | CGGAACGCAAAGCTGTCAGG | 20 |  |
|  |  | LF-probe | BHQ2-ACCCTACCGGTGTTGGTGG-Cy5 | 19 | This study |
| EEHV5 | EEHV5-set1 | F3 | GGTTTGTAAAACCTCATATACGT | 23 | This study |
|  |  | B3 | CATGCCTCTGGAAACACC | 18 |  |
|  |  | FIP | TGTTTTAACTTTTCTCTCACGGCTTTATTCTCTCTCAACTTCTTACATCC | 50 |  |
|  |  | BIP | TGTAGTGACCCTTTGATGCAGATTGGTAAATCCATATACAGCGTTA | 46 |  |
|  |  | LB | CTCGACAAACAGCAGCTTGCC | 21 |  |
|  | EEHV5-set2 | F3 | CAGATTCTACTCGACAAACAG | 21 | This study |
|  |  | B3 | GGCGATATGTCAACTAGAAAGT | 22 |  |
|  |  | FIP | ACGGAAACATGCCTCTGGAAAGCCCTGAAACTTACGTGTA | 40 |  |
|  |  | BIP | TATAGCTGAATCGGTTACCGCCCCAATCGTTAAATCTGTCACAT | 44 |  |
|  |  | LF | ACCGGTAAATCCATATACAGCGT | 23 |  |
|  |  | LB | GGAAGACAGTTGCTAGCCAC | 20 |  |
|  |  | LF-probe | BHQ1-ACCGGTAAATCCATATACAGCGT-FAM | 23 |  |
|  |  | LB-probe | BHQ1-GGAAGACAGTTGCTAGCCAC-FAM | 20 |  |
|  | EEHV5-set3 | F3 | GGATGATGACGTTACGACT | 19 | This study |
|  |  | B3 | GGTAAATCCATATACAGCGTTAC | 23 |  |
|  |  | FIP | CCAGGATGTAAGAAGTTGAGAGAGAGTTACGACTAACACGGGAA | 44 |  |
|  |  | BIP | AAGCCGTGAGAGAAAAGTTAAAACATAAGTTTCAGGGCAAGCT | 43 |  |
|  |  | LF | GAGGTTTTACAAACCTGTGCTTTC | 24 |  |
|  |  | LB | TGTAGTGACCCTTTGATGCAGA | 22 |  |
|  |  | LB-probe | BHQ1-TGTAGTGACCCTTTGATGCAGA-FAM | 22 |  |

**Supplemental Table 2.** The information of the primers used in the qPCR assay.

| Primer sets | Primer name | Primer sequence (5'-3') | Length (nt) | Refs. |
| --- | --- | --- | --- | --- |
| EEHV1 | EEHV1 F | ACTGCAAAYGCATTCTTAAAAGAT | 24 | [2] |
|  | EEHV1 R | AGAATGGGATTRGCTAAGAAGCT | 23 |  |
|  | EEHV1-Probe | CY5-TCAACGAGGAGATATTAGGCACCACCAACA-BHQ2 | 30 |  |
| EEHV5 | EEHV5 F | TGTGACAGATTTAACGATTGGA | 22 | [3] |
|  | EEHV5 R | CTGTCGGTATCACCGTATACTA | 22 |  |
|  | EEHV5-Probe | Fam-CTATACAGAACTTTCCACTATCGACA-BHQ1 | 27 |  |

**Supplemental Table3.** Comparison between the duplex real-time LAMP method and the qPCR method with clinical samples.

|  |  | **qPCR** | | **Total** |
| --- | --- | --- | --- | --- |
|  |  | + | - |  |
| **Duplex real-time LAMP(EEHV1)** | + | 3 | 0 | 3 |
|  | - | 0 | 19 | 19 |
| **Total** |  | 3 | 19 | 22 |
| *Cconcordance rates =22/22=100%* | | | | |

**
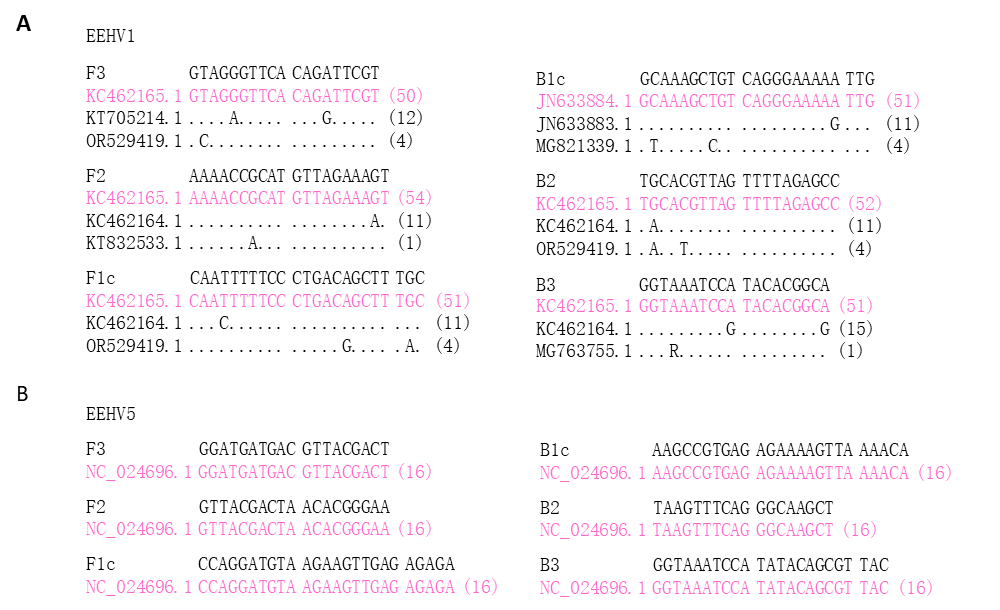
**

**Supplementary Figur1. Sequence alignments of the primer binding regions of EEHV1(A) and EEHV5 (B).** A total of 66 EEHV1 and 16 EEHV5 sequences were downloaded from National Center for Biotechnology Information (NCBI) (https://www.ncbi.nlm.nih.gov/) on March, 2025. The sequence number of each unique sequence/variant is shown in parenthesis. Dot, identity with the topmost sequence.


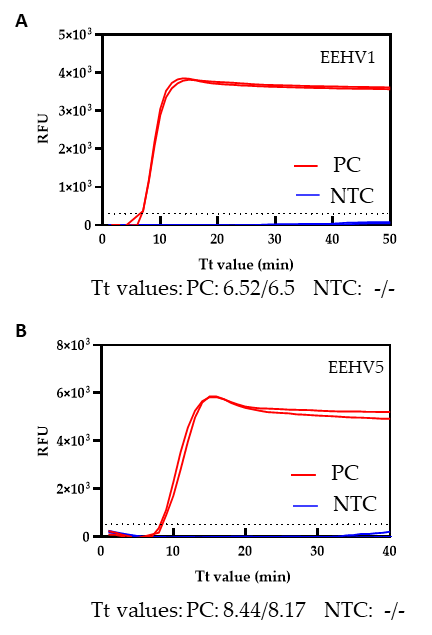


**Supplementary Figur2. Performance of the single real-time LAMP assay in the detection of EEHV1 and EEHV5.** NTC: non-template control.


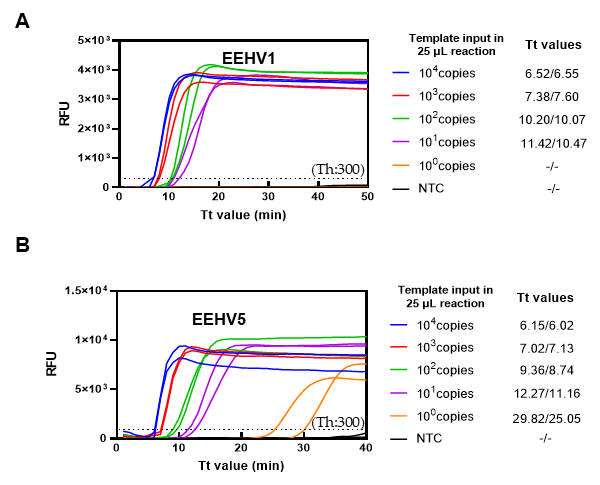


**Supplementary Figure 3. Sensitivity of the single realtime LAMP assay for EEHV1 (A) and EEHV5 (B).** NTC: non-template control.


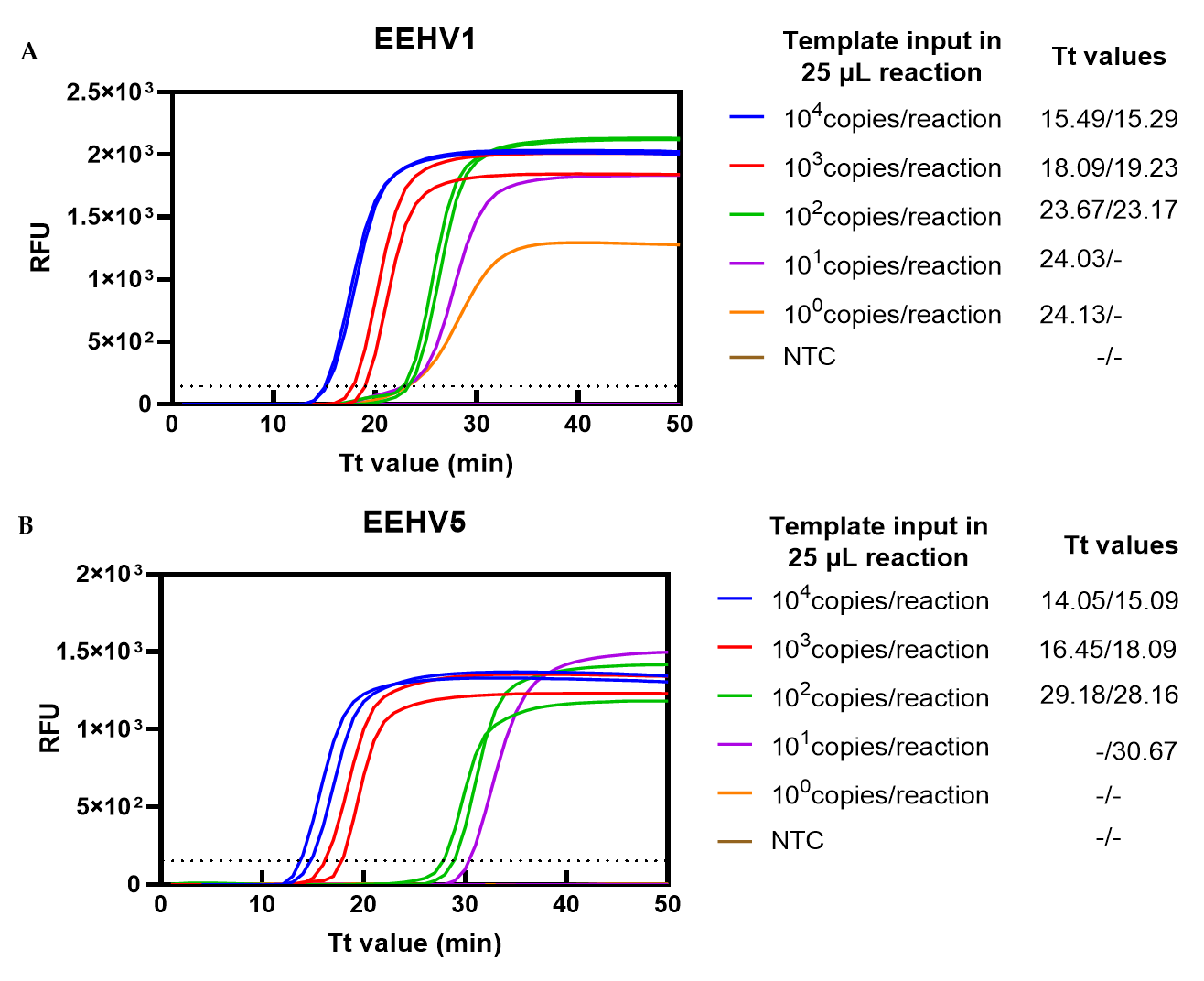


**Supplementary Figure 4. Sensitivity of the duplex real-time LAMP assay for EEHV1 (A) and EEHV5 (B)** (Repeated experiment). NTC: non-template control; Th: fluorescence threshold.

**Supplementary References**

1. Takehana, K.; Kinjyo, T.; Nemoto, M.; Matsuno, K. Rapid and sensitive detection of elephant endotheliotropic herpesvirus 1 (EEHV1) in blood by loop-mediated isothermal amplification (LAMP). *J Vet Med Sci* **2019**, *81* (3), 504-507. DOI: 10.1292/jvms.18-0683

2. Hardman, K.; Dastjerdi, A.; Gurrala, R.; Routh, A.; Banks, M.; Steinbach, F.; Bouts, T. Detection of elephant endotheliotropic herpesvirus type 1 in asymptomatic elephants using TaqMan real-time PCR. *Vet Rec* **2012**, *170* (8), 205. DOI: 10.1136/vr.100270

3. Abdelgawad, A.; Nascimento, M.; Prahl, A.; Flugger, M.; Szentiks, C.A.; Holtze, S.; Hildebrandt, T.B.; Trimpert, J. Fatal infection caused by a genetically distinct elephant endotheliotropic herpesvirus type 5 in a captive Asian elephant in Germany. *Virol J* **2024**, *21* (1), 221. DOI: 10.1186/s12985-024-02477-w
